# Gas dissection from the thorax to the abdomen

**DOI:** 10.36416/1806-3756/e20250184

**Published:** 2025-11-19

**Authors:** Marina Manica Tamiozzo, Letícia Dalmolin, Mariana Manica Tamiozzo

**Affiliations:** 1. Universidade Federal de Santa Maria, Santa Maria (RS), Brasil.; 2. Departamento de Radiologia e Diagnóstico por Imagem, Hospital Universitário de Santa Maria, Universidade Federal de Santa Maria, Santa Maria (RS), Brasil.

**Figure 1:**
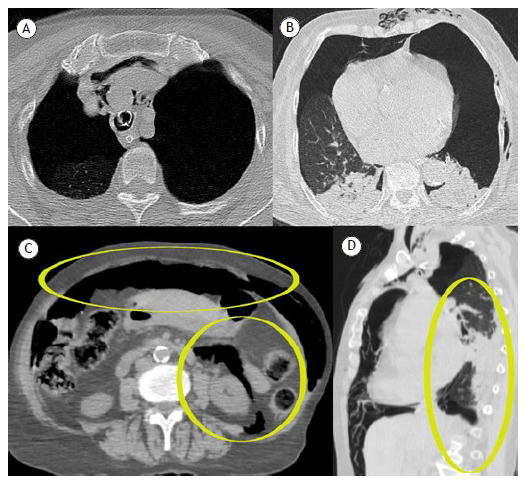
Pneumomediastinum (A) and large bilateral pneumothorax and emphysema on chest CT (B). Pneumoperitoneum and retroperitoneum on abdominal CT scan ellipses (C), and gas dissection of the thorax and infradiaphragmatic gas on sagittal CT slice (D).

An 80-year-old woman with systemic arterial hypertension was admitted to the emergency department after a transient loss of consciousness. On arrival, she presented with hypoxemia and decreased sensorium, requiring orotracheal intubation. Chest computed tomography (CT) revealed a large bilateral pneumothorax, pneumoperitoneum, and retroperitoneal air (Figure 1). A chest tube was placed. An initial suspicion of hollow viscus perforation was ruled out after exploratory laparotomy showed no visceral injury. The combination of imaging findings and clinical context strongly suggested barotrauma secondary to excessive positive-pressure ventilation. This likely resulted in alveolar rupture due to elevated intrathoracic pressure, with air dissecting along bronchovascular sheaths-a phenomenon known as the Macklin effect. From the lungs, air extended into the mediastinum and, in rare cases, progressed as gas dissection through mediastinal vessels into the retroperitoneal space and peritoneal cavity, leading to pneumoperitoneum without visceral perforation. The patient subsequently developed an ischemic stroke and died following clinical deterioration.

Pulmonary barotrauma is uncommon but potentially life-threatening, resulting from sudden increases in intrathoracic pressure. Common complications, such as hypoxemia and subcutaneous emphysema, are typically managed conservatively. However, gas dissection into the retroperitoneum and peritoneum, though rare, represents a serious and diagnostically challenging condition. Early recognition through CT is essential for timely and appropriate management.^1^ ^-^ ^2^
